# Prevalence and risk factors of delirium in psychogeriatric outpatients

**DOI:** 10.1002/gps.5413

**Published:** 2020-09-02

**Authors:** Daisy W. P. Quispel‐Aggenbach, Esther P. R. Schep‐de Ruiter, Wilma van Bergen, J. Rob Bolling, Sytse U. Zuidema, Hendrika J. Luijendijk

**Affiliations:** ^1^ University of Groningen, University Medical Center Groningen, Department of General Practice and Elderly Care Medicine Groningen The Netherlands; ^2^ Parnassia Groep/ Bavo Europoort, Department of Elderly Psychiatry Rotterdam The Netherlands

**Keywords:** delirium, elderly, older patient, prevalence, risk factors

## Abstract

**Background:**

Delirium is a serious neuropsychiatric syndrome, which requires timely treatment. However, it is easily missed, especially in older patients with premorbid cognitive disorders.

**Objectives:**

The aim of this study is to investigate the prevalence and risk factors of delirium in older outpatients with and without dementia.

**Method:**

We assessed 444 patients referred to the memory clinic of a psychiatric hospital between March 2013 and March 2014. Demographic information, medical history, impairments in daily living activities and referral information were registered. Patients underwent a psychiatric examination using the Delirium Rating Scale‐Revised‐98 and cognitive tests, a physical examination and laboratory tests. We recorded medication use and changes before and after the onset of symptoms.

**Results:**

Among the 444 outpatients, 85 had probable delirium (prevalence of 19%), and 10 had subsyndromal delirium (2%). The most common triggers were infection (42%), drug‐intoxication or withdrawal (22%), and metabolic/endocrine disturbance (12%). Age (OR 1.07, 95% CI 1.02‐1.11) and prior delirium (OR 3.34, 95% CI 1.28‐8.69) were independent non‐modifiable factors associated with an increased risk of delirium. The only independent modifiable risk factor was infection (OR 17.31, 95% CI 8.44‐35.49).

**Conclusions:**

A delirium was detected in one of five patients referred for dementia screening. Most patients could be treated at home. Age and prior delirium were predictive of an increased risk of delirium.

**Key points**

- The prevalence of probable delirium in older outpatients with and without dementia was 19%, the prevalence of possible delirium 2%.
- In our study, most patients had a rather mild presentation of delirium and relatively “innocent” underlying illness, which could make delirium hard to detect.
- Infection, drug‐intoxication or ‐withdrawal, urine tract infection, and metabolic/endocrine disturbance had triggered delirium in more than 75% of the study participants; most triggers could be treated ambulatory.
- We recommend the use of a validated screening tool such as the delirium caregiver questionnaire or validated diagnostic tool such as the DRS‐R‐98 to improve the detection of delirium in psychogeriatric outpatients.

## 1 INTRODUCTION

Delirium is a common and serious neuropsychiatric disorder with potentially severe consequences such as poor cognitive and functional recovery. Other consequences are longer hospital stay, increased risk of nursing home placement and death.^1^ Delirium occurs in 10% to 40% of hospitalized and institutionalized patients.^2^, ^3^ Older patients with premorbid cognitive impairment are particularly vulnerable and outcomes of treatment are poor.

It is not recognized widely that delirium occurs in home‐dwelling patients too. Very low prevalences reported in the earliest studies corroborated this view. The first study reported a prevalence of <1% in a general American population of 55 years and older.^4^ Another study in a general Spanish population aged 70 and older found a prevalence of 1%.^5^ In these studies, delirium was a secondary diagnosis based on symptoms that were assessed to establish dementia diagnoses. However, recent studies have shown that delirium is common in frail older patients at home. Two studies reported a prevalence ranging between 16% and 19% in patients of memory clinics of psychiatric hospitals in the Netherlands and Japan.^6^, ^7^ A Scandinavian study among very old patients receiving home care reported an even higher prevalence of 24%.^8^

Around one third of delirium cases go undetected, and proper treatment might be delayed. One reason may be that the symptoms of delirium overlap with those of dementia and depression (2). A history of psychiatric disease might hinder recognition too.^9^ It is also possible that older patients living at home have a rather mild presentation of delirium and relatively “innocent” underlying illness, which make delirium hard to detect.^7^

Risk factors of delirium have mainly been studied in hospitalized patients. The commonest factors significantly associated with delirium were dementia, older age, co‐morbid illness, severity of medical illness, infection, “high‐risk” medication use, diminished activities of daily living, immobility, sensory impairment, urinary catheterization, urea and electrolyte imbalance, and malnutrition.^10^ Another study found that heart disease was a risk factor.^11^ Risk factors of delirium in outpatients have been studied in just a few studies. These studies reported that infections, stressful events, surgery, medical illnesses, heart failure, metabolic‐endocrine disturbances, and the use of medication like benzodiapines and haldoperidol, as well as polypharmacy were associated with an increased risk of prevalent delirium.^7^, ^12^

In hospitalized patients, risk factors are mostly divided in predisposing (contributory) and precipitating (triggering) factors.^13^ This distinction cannot easily be applied to community‐dwelling older patients with delirium. Factors such as dehydration or poorly regulated diabetes can be predisposing and precipitating factors at the same time, and occur relatively frequently in older patients. Therefore, we prefer to distinguish non‐modifiable and modifiable risk factors. Studies about the risk factors of delirium in patients at home are needed to guide doctors in diagnosis and treatment.

The aim of our study was to assess the prevalence as well as non‐modifiable and modifiable risk factors of delirium in older outpatients with and without dementia.

## 2 MATERIALS AND METHODS

### 2.1 Design and participants

We performed a study among older patients consecutively referred for dementia screening to an outpatient clinic of a psychiatric institution between March 2013 and March 2014. Patients resided in and around Rotterdam, the Netherlands. Most of them were referred by their general practitioner, who provides primary care to older patients who live at home or in care centers. Some patients were referred by a geriatrician for extended psychiatric treatment after hospitalization. The patients had cognitive disorders with or without psychological or behavioral disorders, and some were suspected of having delirium at the time of referral. Patients referred for the second time during the study period were only included at the time of the first referral. We excluded hospitalized and institutionalized patients who were referred for consultation, because these patients receive medical care from other specialists than general practitioners.

The Medical Ethics Committee of the Erasmus University of Rotterdam, The Netherlands, approved the study protocol. The committee granted a waiver of consent for patients, because data were collected as part of (enhanced) daily medical care. The study did not pose a risk to the patient.

### 2.2 Measurements

A geriatrician and a registered psychiatric nurse assessed the patients. They visited the patients one to three times in their (care) home (not nursing home). An informal caregiver was usually present.

To establish a delirium diagnosis, we interviewed the patient and his caregiver, performed a psychiatric and physical examination, and ordered standardized blood‐ and urine tests. Cognitive functioning was tested with the Mini‐Mental State Examination and the Clock Drawing Test.^14^, ^15^ Given the fluctuating course of delirium, we interviewed caregivers in detail about the presence and onset of neuropsychiatric symptoms and fluctuations in cognitive, behavioral, and physical functioning during and across days.

For every patient the medical file was checked for symptoms of delirium and possible underlying somatic illnesses. If necessary, we obtained additional information from the general practitioner, hospital‐based specialists and home care reports. We recorded medications used and changes in medication in the weeks prior to delirium onset. If necessary, the general practitioner could order additional tests such as a urine culture test or an X‐ray of the thorax.

### 2.3 Delirium diagnosis

We recorded symptoms and severity of delirium from the first occurrence until intake on the Delirium Rating Scale‐Revised‐98.^16^ The DRS‐R‐98 is divided in two parts. The first 13 items refer to the symptoms of delirium and the last three items to the diagnostic criteria of delirium (acute onset, fluctuations, somatic illness). We used this scale to structure and standardize our psychiatric assessment. Our team did not use the DRS‐R‐98 before we started the study.

The final diagnosis of probable delirium was based on the criteria for delirium outlined in the Diagnostic and Statistical Manual of Mental Disorders (DSM‐IV‐TR).^17^ If one criterion was not met, we diagnosed a subsyndromal delirium. A diagnosis of dementia, on which delirium could be superimposed, was also based on DSM‐IV‐TR criteria. If patients with delirium had cognitive decline before onset of delirium but no formal diagnosis of dementia, the diagnosis of dementia was postponed until the delirium had remitted and the cognitive situation of the patient could be re‐evaluated. For this evaluation, we obtained anamnestic and hetero‐anamnestic information and tested the patient with the MMSE and the clock‐drawing test.

### 2.4 Non‐modifiable and modifiable risk factors

First, we extracted referral data from the medical file: reason for referral (cognitive screening, counseling, and treatment for cognitive disorders with or without psychological or behavioral disorders), request for an emergency visit at the time of referral (within at most 2 days), hospitalization within 3 months before referral, and place of residence (own home, or care [not nursing] home).

We registered the following non‐modifiable risk factors: age, sex, medical history including prior delirium (no/yes), prior dementia (none, dementia, other cognitive disorder), and polypharmacy (mean number of drugs, and use of five drugs or more), as well as impairments including impaired hearing (no/slight limitation/yes), impaired sight (no/slight limitation/yes), level of activities of daily living (ADL) on the Katz‐scale^18^ (range 0‐6, higher is worse), and impaired walking inside (no/yes).

Finally, underlying disorders were recorded in plain text by the attending physicians. We extracted these modifiable risk factors from the patient files and categorized them as: drug‐intoxication or withdrawal, metabolic/endocrine disorder, infection (intra‐cranial or systematic), neoplasm (intra‐cranial or systematic), cerebrovascular attack, heart disease, and other disease. The last category included for example head trauma, pain, constipation, and major surgery. This categorization has been introduced in a previous study in a similar outpatient population and we deemed it very suitable for our study.^7^ We recorded which disorder had been the likely trigger of delirium; there could be more than one trigger per delirium case.

### 2.5 Statistical analysis

First, we used descriptive statistics to describe patients with probable delirium, patients with possible delirium, and patients without delirium. For every group we calculated means for continuous data, and percentage for binary data. We used the independent sample *t*‐test for means and Chi‐square test for binary data to determine statistical significance (*P* < .05).

Next, we investigated the relationship of non‐modifiable and modifiable risk factors with the presence of delirium with logistic regression. Our preference was to include the following factors and potential confounders in the model: age, sex, impaired hearing, impaired sight, prior delirium, prior dementia (diagnosis before intake), drug‐intoxication or withdrawal, metabolic/endocrine disorder, infection, neoplasm, cerebrovascular attack, and heart failure. However, the absolute number of delirium cases eventually forced us to limit the number of variables to six or seven.^19^ Moreover, univariate analyses yielded virtually the same odds ratios as our complete model. Therefore, we ran a correlation analysis and it showed that age was significantly correlated with sex and impaired hearing, and impaired hearing with impaired sight. Hence, our final model included the factors associated with delirium in the univariate model (age, impaired hearing, prior delirium, infection) and the correlated factors (age, sex, impaired hearing, impaired sight). We coded the modifiable risk factors as present whether or not they were considered a trigger for the current delirium. We excluded subsyndromal delirium in this analysis to avoid loss of power due to uncertainty around this diagnosis. We used stata 15 for data analysis.^20^

## 3 RESULTS

### 3.1 Study population

**FIGURE 1:**
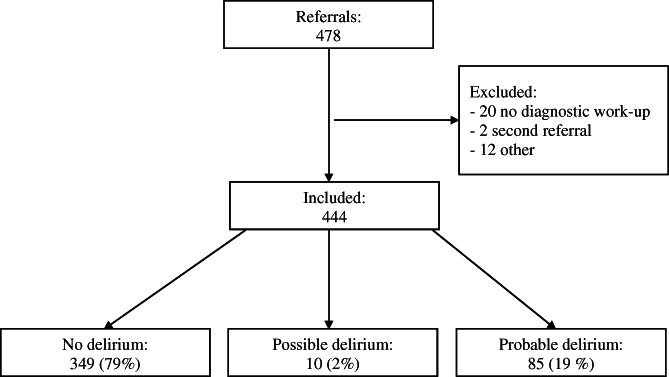
Flow diagram of participants

During the study period, 478 patients were referred to our outpatient clinic for psychogeriatric assessment and treatment, of which 444 patients were included in our study (see flow diagram Figure 1). Thirty‐four patients were excluded. Twenty patients did not receive a diagnostic work‐up because they did not want to take part in a diagnostic assessment, discontinued the diagnostic assessment, or died during the assessment period. Two patients had been referred for the second time in the study period. For another 12 institutionalized or hospitalized patients, only a one‐time consultation was asked.

### 3.2 Prevalence of delirium

Among the 444 patients who received a diagnostic work up, there were 309 women (70%) and 135 men (30%). The mean age was 82.9 years. The referral reasons were cognitive screening (285/64%), counseling and treatment for cognitive disorders with or without psychological or behavioral disorders (159/36%), and suspected delirium (9/2%).

Of the 444 patients, 85 had probable delirium (prevalence of 19%), and 10 patients had subsyndromal/possible delirium (prevalence of 2%). Of the nine patients referred for suspected delirium, eight had probable delirium. The factors that had triggered delirium in more than 75% of patients were infection (36/42%), drug‐intoxication or withdrawal (19/22%), and metabolic/endocrine disturbance (10/12%).

### 3.3 Risk factors

Table 1 shows the patient characteristics for patients without delirium, with possible delirium and with probable delirium. The patients with probable delirium were older than the patients without delirium (86.6 vs 82.1 years, respectively), lived in a care center more often (28% vs 12%), and had more impaired ADL (3.0 vs 1.9).

**TABLE 1:** Patient characteristics

	No delirium (n = 349)	Possible delirium (n = 10)	Probable delirium (n = 85)
*Demographic characteristics*			
Age, mean (SD)	82.1 (8.6)	83.2 (3.1)	86.6 (7.5)**
Sex, n (%)			
Male	108 (31)	5 (50)	22 (26)
Female	241 (69)	5 (50)	63 (74)
Place of residence, n (%)			
home	306 (88)	6 (60)*	61 (72%)**
care centre	43 (12)	4 (40)*	24 (28%)**
ADL, mean (SD)	1.9 (1.6)	2.3 (1.9)	3.0 (1.4)**
Walking inside impaired, n (%)	108 (31)	6 (60)	45 (53)**
*Medical history*			
History of delirium, n (%)	15 (4)	2 (20)*	15 (18)**
History of dementia^a^, n (%)			
Dementia, any type	25 (7)	3 (30)	6 (7)
Other cognitive disorders	48 (14)	6 (60)	11 (13)
No cognitive disorder	276 (79)	1 (10)	68 (80)
Impaired hearing, n (%)	94 (27)	5 (50)	34 (40)*
Impaired sight, n (%)	109 (31)	1 (10)	26 (31)
Number of medication			
Mean (SD)	5.7 (3.9)	6.5 (3.7)	7.1 (3.9)*
Five or more medicines, n (%)	198 (58)	7 (70)	63 (76)*
Recent hospital discharge, n (%)	18 (5)	0 (0)	27 (32)**
Emergency visit requested, n (%)	13 (4)	0 (0)	27 (32)**
*Modifiable risk factors*			
Drugs, n (%)	316 (92)	10 (100)	82 (96)**
Probable trigger	0 (0)	0 (0)	19 (22)
Metabolic/endocrine disorder, n (%)	173 (50)	7 (70)	46 (54)**
Probable trigger	0 (0)	3 (30)	10 (12)
Infection, n (%)	15 (4)	2 (20)*	40 (47)**
Probable trigger	0 (0)	0 (0)	36 (42)
Neoplasm, n (%)	23 (7)	2 (20)	6 (7)**
Probable trigger	0 (0)	0 (0)	4 (5)
Cerebrovascular attack, n (%)	84 (24)	4 (40)	20 (24)*
Probable trigger	0 (0)	0 (0)	2 (2)
Heart, n (%)	88 (26)	10 (100)	25 (29)**
Probable trigger	0 (0)	0 (0)	5 (6)
Other illness, n (%)	80 (23)	2 (20)	20 (24)**
Probable trigger	0 (0)	0 (0)	4 (5)

^a^ Dementia diagnosis before intake. *P*‐values for (possible/ probable) delirium vs no delirium:

^*^ <.05.

^**^ <.01.

In addition, patients with delirium had a history of delirium more often (18% vs 4%) and polypharmacy (76% vs 58%). Twenty‐seven patients with delirium had been recently discharged from hospital (32%) vs 18 patients without delirium (5%) and 27 patients (32%) vs 13 patients (4%) were referred to our clinic for an emergency assessment.

Table 2 shows the association between risk factors and risk of delirium. Independent risk factors of delirium in patients referred for cognitive screening were: age (OR 1.07, 95% CI 1.02‐1.11), prior delirium (OR 3.34, 95% CI 1.28‐8.69), and infection (OR 17.31, 95% CI 8.44‐35.49).

**TABLE 2:** Association of risk factors with delirium*

	Univariate model		Multivariate model	
	OR (95% CI)	*P*‐value	OR (95% CI)	*P*‐value
*Non‐modifiable risk factors*				
Age in years	*1*.*07 (1*.*03‐1*.*12)*	.001	*1*.*07 (1*.*02‐1*.*11)*	.002
Female sex	0.72 (0.37‐1.41)	.338	0.83 (0.47‐1.58)	.574
Impaired hearing	1.47 (0.73‐2.95)	.276	1.53 (0.78‐3.01)	.220
Impaired sight	0.91 (0.45‐1.83)	.793	0.91 (0.46‐1.80)	.786
History of dementia**				
Dementia (any type)	0.70 (0.29‐1.69)	.422	**‐**	**‐**
Other cognitive disorder	1.00 (0.32‐3.14)	.995	**‐**	**‐**
History of delirium	3.46 (1.28‐9.34)	.014	3.34 (1.28–8.69)	.013
*Modifiable risk factors* ^a^				
Drug intoxication/withdrawal	2.05 (0.50‐8.40)	.317	‐	‐
Metabolic/endocrine disorders	1.50 (0.82‐2.72)	.185	‐	‐
Infection	20.18 (9.40‐43.32)	.000	17.31 (8.44‐35.49)	.000
Neoplasm	1.07 (0.34‐3.36)	.911	‐	‐
Cerebrovascular attack	1.06 (0.53‐2.11)	.873	‐	‐
Heart disease	0.65 (0.32‐1.32)	.233	‐	‐

^a^ Included as present whether or not considered a trigger of delirium.

^*^ Probable delirium only.

^**^ Dementia diagnosis before intake.

## 4 DISCUSSION AND CONCLUSION

We studied the prevalence and risk factors of delirium in 444 older outpatients referred for cognitive screening. The prevalence of probable delirium was 19% and 2% had a subsyndromal delirium. The triggers were most often infection, drug‐intoxication or withdrawal, and metabolic/endocrine disturbance. Age and prior delirium were statistically significant non‐modifiable risk factors of an increased risk of delirium, as was the modifiable risk factor infection.

### 4.1 Prevalence of delirium

The prevalence of 19% for probable delirium, which we found, was very close to that in two other studies in memory clinics of psychiatric institutions. One study found a prevalence of 16% among Dutch patient with a mean age of 86 years,^7^ the other study a prevalence of 19% among Japanese older patients with a mean age of 81 years.^6^ These studies also applied the DRS‐R‐98. The prevalences fall within those of 10% to 40% established in hospitals, nursing homes, and older patients receiving home care.^2^, ^3^, ^8^ In contrast, only studies in general (non‐frail) older populations that did not use a diagnostic tool for delirium reported prevalences below 1%.^4^, ^5^

Our and prior findings show that physicians need to be alert to delirium in older patients who are frail, ill, receive daily home care or have cognitive disorders. Nevertheless, delirium in older patients at home is underdiagnosed and often mistaken for dementia or other psychiatric diseases.^6^ Adequate detection of delirium starts with the recognition that delirium is common in certain subgroups of older patients living at home. Screening frail older persons regularly with a validated screening tool for delirium may help detect delirium more quickly and has been advised in guidelines.^21^, ^22^ However, the existing instruments take a lot of time to administer, and are not very suitable for triage in outpatient settings, and not very sensitive for delirium in dementia.^7^, ^23^ The delirium caregiver questionnaire is a short instrument specifically developed for triage in older outpatients referred for cognitive screening.^24^ Use of the tool has been shown to expedite the detection of delirium. In addition, the use of a validated diagnostic tool for delirium that requires the assessors to perform a detailed interview and examination may help to increase detection rates.

We used the DRS‐R‐98 in the current study and the rate of identified probable delirium increased from around 3% prior to the study to 19% during the study. The addition of the DRS‐R‐98 was the only change made to the assessment protocol for the purpose of the study. Experienced physicians and psychiatric nurses can implement the DRS‐R‐98 fairly easily, because the tool only structures the work they already do and it is not administered to the patient. The CAM is popular for training general nurses but may lack sensitivity in patients with dementia.^25^

### 4.2 Risk factors

The non‐modifiable risk factors that we identified confirm those mentioned in an earlier Dutch study among outpatients referred for dementia screening. In this study, a quarter of the patients had a history of delirium, and half had multiple physical disorders or a hospital stay in the last three months before referral.^7^ These factors indicate an a‐priori susceptibility to delirium or the presence of potential underlying triggering diseases. To our knowledge, no other study has investigated risk factors of delirium in older outpatients.

We found that a diagnosis of dementia before intake was not significantly related to an increased risk of delirium at diagnosis, even though dementia is a well‐known risk factor for delirium.^26^ Given the long lag between onset and diagnosis of dementia, some cases of prior dementia have been misclassified in our study population. This could not be resolved by including diagnoses at intake, because doctors are reluctant to diagnose dementia in patients with delirium in line with good clinical practice.

The circumstances and health status of patients living at home are different than patients with delirium in a hospital. Most modifiable risk factors such as drug‐intoxication and urinary tract infections were relatively easy to treat. Often, patients had two or more triggering diseases. We were able to treat almost every patient at home, and admission to a hospital or nursing home was avoided. Of course, providing regular check‐ups throughout the day and the absence of danger for the patient and other persons was elemental.

### 4.3 Strengths and limitations

Our study provides information about delirium in older outpatients—a topic that has so far been under‐researched. A methodological strength of our study was the high number of included patients, which enhances precision of the estimated prevalence and effects of risk factors. In addition, experienced psycho‐geriatricians and psychiatric nurses performed the assessments. They used the DRS‐R‐98 to structure their psychiatric assessment. This tool has good diagnostic qualities and inter‐rater reliability and covers many different symptoms of delirium. The DRS‐R‐98 was used in other major studies researching delirium, which enables comparison of results.^6^, ^7^

We did not exclude patients based on co‐morbid diseases such as dementia or psychiatric disease, as other studies did, to maintain generalizability.^27^, ^28^ Nevertheless, the prevalence of delirium of 19% might not be applicable to memory clinics in general hospitals. It is likely that patients with psychiatric symptoms such as hallucinations, delusions, and affect lability, which are distinctive of delirium might be referred more often to the outpatient clinic of a psychiatric hospital, whereas patients without psychiatric symptoms might be referred to neurological outpatient clinics.

### 4.4 Conclusion

We found that almost one in five patients referred for dementia screening to the memory clinic of our psychiatric institution had delirium. High age, prior delirium, and infection increased the risk of delirium. Most modifiable risk factors such as drug‐intoxication and urinary tract infections were relatively easy to treat. We recommend the standard use of a good diagnostic tool such as the DRS‐R‐98 to increase the number of detected cases of delirium in psychogeriatric outpatients.

## CONFLICT OF INTEREST

None declared.

## AUTHOR CONTRIBUTIONS

Daisy W. P. Quispel‐Aggenbach collected data and supervised data‐collection, performed the statistical analysis and wrote the manuscript. Esther P. R. Schep‐de Ruiter, Wilma van Bergen, and J. Rob Bolling collected data and provided feedback on the manuscript. Sytse U. Zuidema provided feedback on the manuscript. Hendrika J. Luijendijk designed the study, supervised the study and the statistical analysis, and helped write the manuscript.

## ETHICS STATEMENT

The medical ethics committee of Erasmus Medical Center Rotterdam, an academic research institute that we consulted, has approved the study protocol.

## Data Availability

The data that support the findings of this study are available on request from the corresponding author. The data are not publicly available due to privacy or ethical restrictions.
